# Supplementary figures and images for: Subcellular Localization of Fad1p in Saccharomyces cerevisiae: A Choice at Post-Transcriptional Level?
Source: Life (Basel). 2021 Sep 14;11(9):967. doi: 10.3390/life11090967 (PMC8470081; doi:10.3390/life11090967)

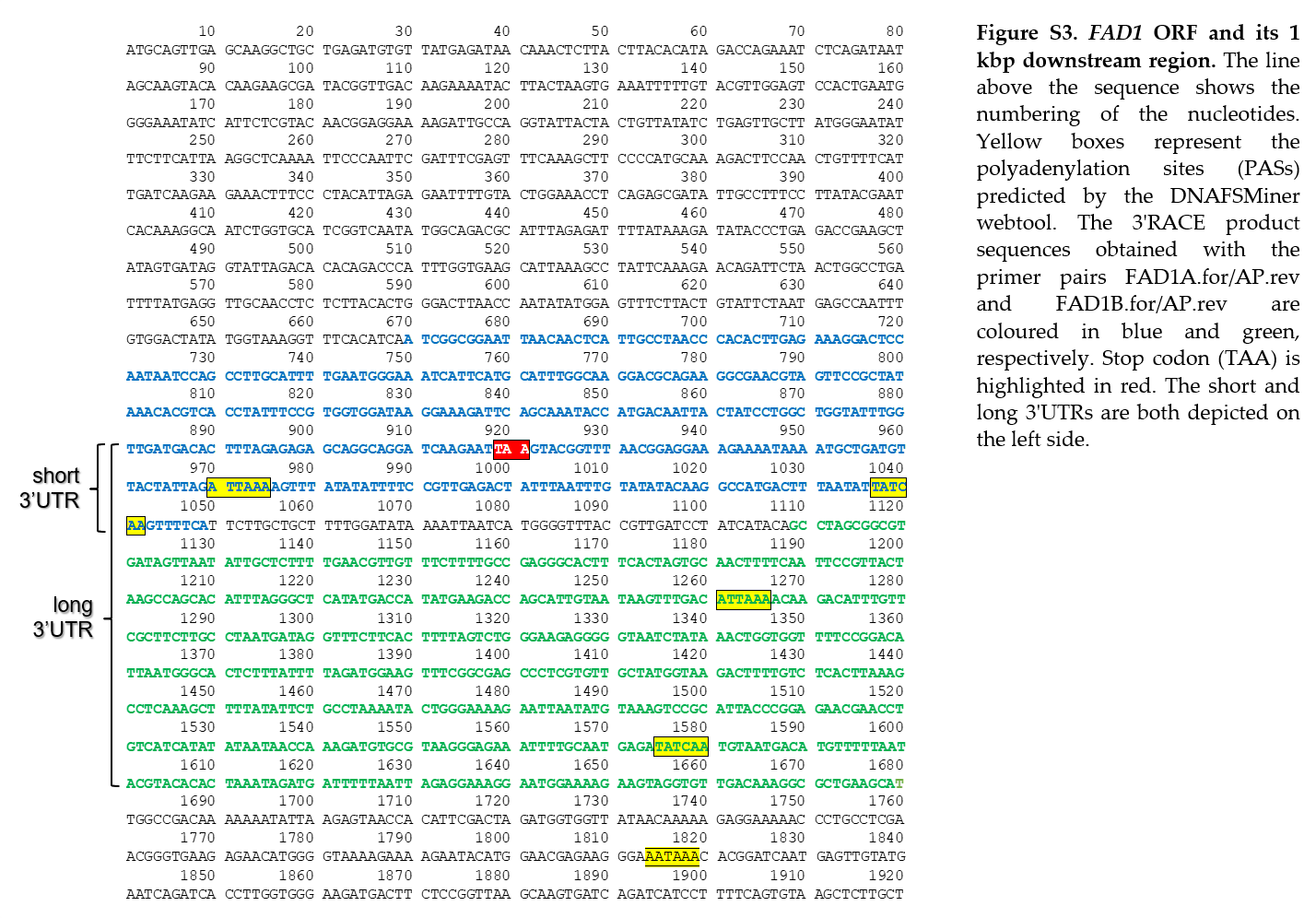

Supplement: Supplementary file 1 [file life-11-00967-s001.zip › Figure S3 + legend.tif]

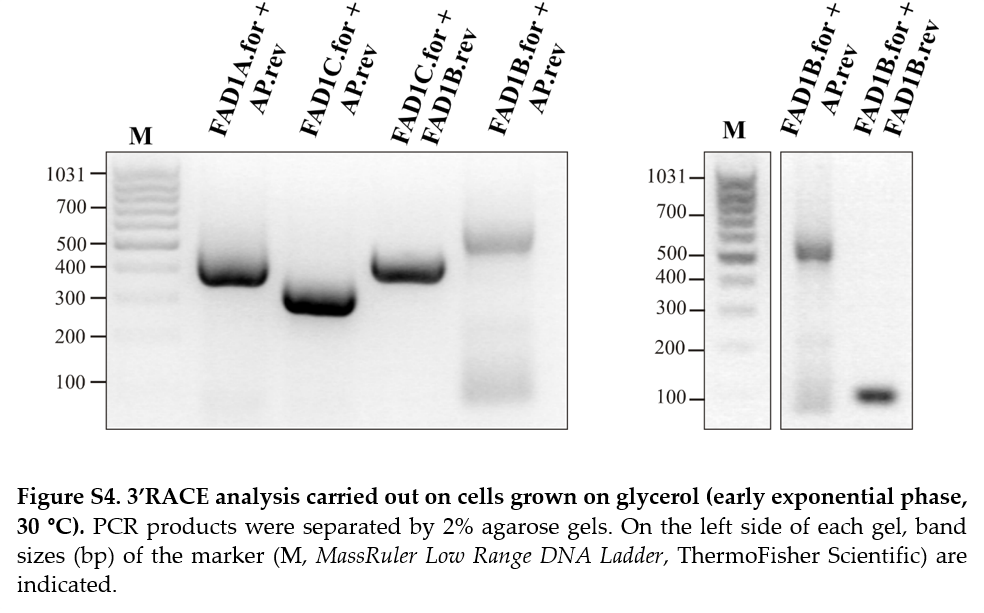

Supplement: Supplementary file 1 [file life-11-00967-s001.zip › Figure S4 + legend.tif]

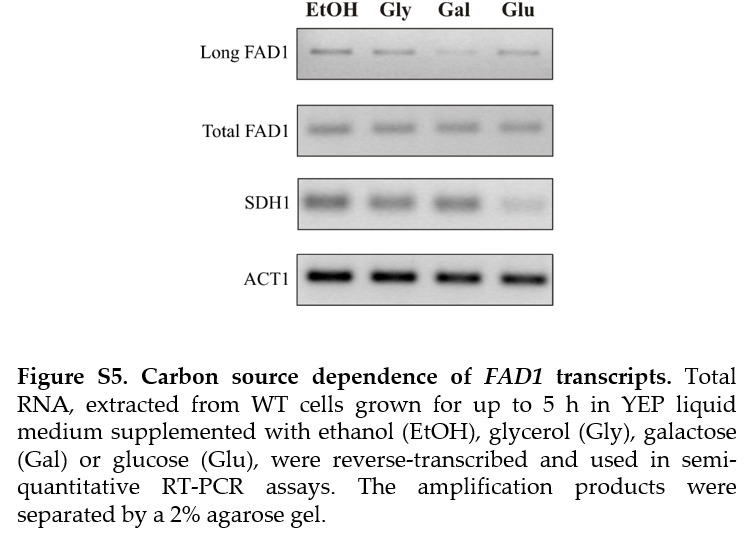

Supplement: Supplementary file 1 [file life-11-00967-s001.zip › Figure S5 + legend.tif]

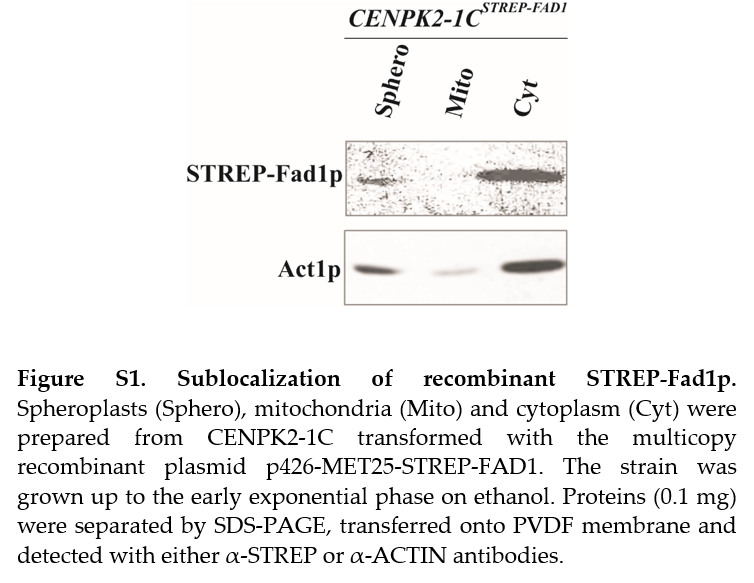

Supplement: Supplementary file 1 [file life-11-00967-s001.zip › Figure S1 + legend.tif]

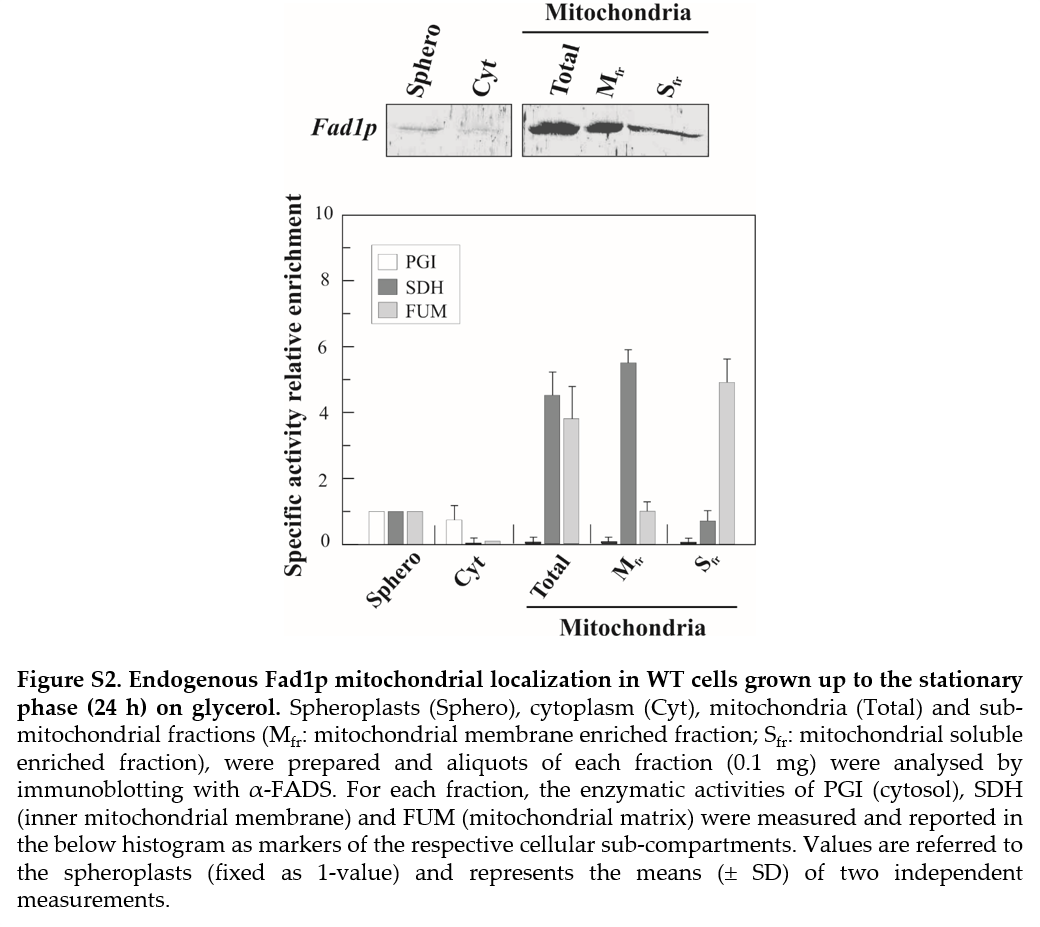

Supplement: Supplementary file 1 [file life-11-00967-s001.zip › Figure S2 + legend.tif]
